# The Protective Role of Heme Oxygenase-1 in Atherosclerotic Diseases

**DOI:** 10.3390/ijms20153628

**Published:** 2019-07-24

**Authors:** Yoshimi Kishimoto, Kazuo Kondo, Yukihiko Momiyama

**Affiliations:** 1 Endowed Research Department “Food for Health”, Ochanomizu University, 2-1-1 Otsuka, Bunkyo-ku, Tokyo 112-8610, Japan; 2 Institute of Life Innovation Studies, Toyo University, 1-1-1 Izumino, Itakura-machi, Ora-gun, Gunma 374-0193, Japan; 3 Department of Cardiology, National Hospital Organization Tokyo Medical Center, 2-5-1 Higashigaoka, Meguro-ku, Tokyo 152-8902, Japan

**Keywords:** heme oxygenase-1, atherosclerosis, coronary artery disease, peripheral artery disease, carotid plaque

## Abstract

Heme oxygenase-1 (HO-1) is an intracellular enzyme that catalyzes the oxidation of heme to generate ferrous iron, carbon monoxide (CO), and biliverdin, which is subsequently converted to bilirubin. These products have anti-inflammatory, anti-oxidant, anti-apoptotic, and anti-thrombotic properties. Although HO-1 is expressed at low levels in most tissues under basal conditions, it is highly inducible in response to various pathophysiological stresses/stimuli. HO-1 induction is thus thought to be an adaptive defense system that functions to protect cells and tissues against injury in many disease settings. In atherosclerosis, HO-1 may play a protective role against the progression of atherosclerosis, mainly due to the degradation of pro-oxidant heme, the generation of anti-oxidants biliverdin and bilirubin and the production of vasodilator CO. In animal models, a lack of HO-1 was shown to accelerate atherosclerosis, whereas HO-1 induction reduced atherosclerosis. It was also reported that HO-1 induction improved the cardiac function and postinfarction survival in animal models of heart failure or myocardial infarction. Recently, we and others examined blood HO-1 levels in patients with atherosclerotic diseases, e.g., coronary artery disease (CAD) and peripheral artery disease (PAD). Taken together, these findings to date support the notion that HO-1 plays a protective role against the progression of atherosclerotic diseases. This review summarizes the roles of HO-1 in atherosclerosis and focuses on the clinical studies that examined the relationships between HO-1 levels and atherosclerotic diseases.

## 1. Introduction

Atherosclerotic diseases are known to be the leading causes of death in the world. Atherosclerosis begins when the injured (or activated) artery wall creates chemical signals that cause certain types of leukocytes to attach to the endothelium [1]. These cells move into the wall of the artery and are transformed into foam cells by uptake of modified low-density lipoprotein (LDL) such as oxidized LDL, which collect cholesterol and other fatty materials and trigger smooth muscle cells to migrate from the media to the intima. They form atheromas, also called plaques, covered with a fibrous cap, and eventually the growing lesion begins to raise the endothelium and encroach on the lumen of the artery. When plaques rupture, the exposing material triggers blood clot formation, which can suddenly block blood flow through the artery, resulting in myocardial infarction or stroke.

Although heme serves key physiological functions and is tightly controlled, high levels of free heme, which may occur in various pathophysiological conditions, are toxic via pro-oxidant, pro-inflammatory, and cytotoxic effects [2]. Thus, the heme degradation pathway has been demonstrated to play a protective role against the development of atherosclerosis [3,4,5]. Heme oxygenase (HO) is the rate-limiting intracellular enzyme that catalyzes the oxidation of heme to generate biliverdin, carbon monoxide (CO), and ferrous iron. Biliverdin is subsequently reduced to bilirubin by biliverdin reductase; both of biliverdin and bilirubin have antioxidant properties. The endogenously produced CO can serve as a second messenger affecting several cellular functions, including proliferation, inflammation, and apoptosis [3,6,7]. Three isoforms in the HO family (HO-1, HO-2, and HO-3) are known to be the products of different genes and to be differently regulated. HO-1, also known as a 32-kDa heat shock protein, encoded by the gene *HMOX1*, is normally expressed at low levels in most tissues; however, HO-1 is highly inducible in response to various stresses/stimuli, including heme/hemoglobin, heavy metals, UV radiation, cytokines, and endotoxins [8,9,10,11,12]. In contrast, HO-2 is constitutively expressed in most tissues. HO-3 has a protein structure that is similar to that of HO-2 but has lower enzymatic activity and is less well characterized [13]. A variety of experimental studies have suggested that HO-1 is a stress-response protein that plays an important role in cell defense mechanisms against oxidative injury. In the pathogenesis of atherosclerosis, the ability of HO-1 to generate biliverdin and bilirubin, anti-oxidant molecules, and CO, a vasodilator and an anti-inflammatory and antiapoptotic molecule, is thought to play important roles in protecting the artery against oxidant-induced injury. This review documents the roles of HO-1 in atherosclerosis and focuses on the clinical significance as a potential therapeutic target in atherosclerotic diseases, such as coronary artery disease (CAD) and peripheral artery disease (PAD). Using a PubMed database, we reviewed the articles published by July 2019 only in English. The clinical studies included in this review that showed the relationships between HO-1 levels and atherosclerotic diseases are summarized in Table 1.

## 2. Important Role of HO-1 in Atherosclerosis

*HMOX1* deficiency is very rare in humans [28,29]. In two reported cases, similar phenotypes characterized by generalized inflammation, nephropathy, asplenia, anemia, and tissue iron deposition were observed. Vascular injury and early atherosclerotic changes, as reflected by the presence of fatty streaks and fibrous plaques were also reported, suggesting the importance of HO-1 in vascular health. In HO-1-knockout (*Hmox1^−/−^*) mice, growth retardation, anemia, iron deposition, and vulnerability to stressful injury were observed [30,31]. *Hmox1*^-/-^ mice were also reported to develop severe aortitis and coronary arteritis with mononuclear cellular infiltration and fatty streak formation even on a standard chow diet [32].

In 1998, Wang et al. demonstrated that HO-1 expression was present throughout the development of human atherosclerotic lesions from early fatty streaks to advanced lesions [14]. Oxidized LDL, a major determinant in the pathogenesis of atherosclerosis, was identified to be a potent inducer of HO-1. The HO-1 expression in endothelial cells, monocytes, and macrophages was up-regulated by exposure to oxidized LDL [33,34,35]. HO-1 expression in atherosclerotic lesions is thus considered to be a protective response against the progression of atherosclerosis. HO-1 overexpression by pharmacological inducers or viral gene transfer successfully inhibited atherogenesis in hypercholesterolemic animal models [36,37,38]. In contrast, the genetic ablation of *Hmox1* in apolipoprotein E-knockout mice accelerated the development of atherosclerosis and exacerbated lesion formation [39]. These results thus suggest that HO-1 plays a protective role against the progression of atherosclerosis.

The genetic polymorphisms of the *HMOX1* gene in humans also indicate the potential importance of HO-1 in the pathogenesis of cardiovascular diseases (CVDs). Among the identified polymorphisms in the *HMOX1* gene, two have attracted the most attention: A (GT)n dinucleotide repeat length polymorphism and a common single-nucleotide polymorphism (SNP), T(-413)A (rs2071746) [40]. The (GT)n short allele (S, <25 repeats) and the A(-413) allele are reported to be associated with significantly increased *HMOX1* gene promoter activity compared to the long allele (L, ≥25 repeats) and the T(-413) allele, respectively [41,42]. The association of these polymorphisms with CAD has been discussed [18,43,44,45,46,47]. A meta-analysis by Qiao et al. [40] demonstrated that the (GT)n SS genotype was associated with a decreased risk of CAD after controlling for biases (age, sex, extent of coronary stenosis, ethnicity, and study quality). For the T(-413)A SNP, although a decreased CAD risk among individuals with the AA genotype was observed compared to individuals with the TT genotype, the authors mentioned that this effect was quite limited and should be interpreted cautiously [40].

## 3. Mechanistic Actions of HO-1 in Oxidative Stress and Inflammation

HO-1 is known to be regulated by the redox-sensitive transcription factor known as nuclear factor erythroid 2-related factor (Nrf2) [48]. Nrf2 is ubiquitously expressed and kept in a latent state through the interaction with its repressor protein, Kelch ECH associated protein 1 (Keap1). The exposure of cells to oxidative stimuli triggers a conformational change in Keap1 through a modification of its cysteine residues, which results in the release of Nrf2 from Keap1. Apart from this Keap1-dependent pathway, Nrf2 activation is also mediated by protein kinases such as glycogen synthase kinase-3β (GSK-3β), phosphatidylinositol-3-kinase (PI3K)/Akt, protein kinase C (PKC), and mitogen-activated protein kinase (MAPK) cascades via the phosphorylation of the serine or threonine residues of Nrf2. Stabilized cytosolic Nrf2 is translocated into the nucleus and binds to the antioxidant response element (ARE), thereby initiating the transcription of antioxidant and phase II detoxification enzymes, including HO-1, superoxide dismutase (SOD), catalase, and NAD(P)H quinone dehydrogenase 1 (NQO1) [49]. Additionally, Nrf2 has demonstrated anti-inflammatory properties through its ability to negatively regulate nuclear factor-kappaB (NF-κB), the transcription factor central to the inflammatory response [50].

The anti-oxidant activity of HO-1 is thought to be due to its byproducts biliverdin, bilirubin, and CO. Bilirubin strongly scavenges several oxygen free radicals including singlet oxygen, O^2 −^, ONOO^−^, and organic peroxy radicals [51,52]. Because of its lipophilic property, bilirubin is closely associated with cell membranes, and hence can protect them against lipid damage and also protect LDL against peroxidation. It was reported that the patients with Gilbert syndrome (i.e., unconjugated hyperbilirubinemia) had lower circulating levels of oxidized LDL [53] and also that total bilirubin levels were associated with oxidized LDL levels [54,55]. On the basis of the involvement of oxidized LDL in the formation of atherosclerotic plaques, it was suggested that increased physiological concentrations of plasma bilirubin may reduce atherogenic risk.

In 1994, Schwertner et al. [56] reported serum bilirubin levels to be low in patients with CAD. Since then, many studies showed bilirubin levels to be lower in patients with CAD than without CAD and to inversely correlate with the severity of CAD [57,58,59]. Low bilirubin levels in blood may thus play a promotive role in the development of CAD. Genetic variations in the UDP-glucuronosyltransferase 1A1 (*UGT1A1*) gene are known to be major determinants of serum bilirubin level [60]. However, Stender et al. [61] investigated the associations between the *UGT1A1* gene genotype and plasma bilirubin levels and between genetically elevated bilirubin levels and CAD in 67,068 subjects. They demonstrated that genetically elevated bilirubin levels were not associated with a decreased risk of CAD. They also performed a meta-analysis of 11 studies and showed no association between genetically elevated bilirubin levels and CAD. These findings thus suggest no causal relationship between elevated bilirubin levels and CAD. Since increased reactive oxygen species (ROS) and oxidative stress are involved in the pathogenesis of atherosclerosis, low bilirubin levels in patients with CAD may not be a cause of CAD but rather a result of increased oxidative stress, leading to the consumption of endogenous anti-oxidants [52].

It is suggested that CO can attenuate the production of intracellular ROS. Kobayashi et al. [62] demonstrated that low-dose exogenous CO exposure inhibited the activation of NADPH oxidase and effectively suppressed ROS generation in the heart tissues of angiotensin II–infused mice. This finding might support the previous in vitro observation that HO-1 inhibited NADPH oxidase activity in cultured cells [63]. Importantly, obesity enhances the activation of NADPH oxidase and the angiotensin II system, resulting in the development of diabetes and hypertension in part due to impairment of adipocyte function [64]. Hinds and colleagues demonstrated that the induction of HO-1 in obese mice resulted in the elevation of peroxisome proliferator-activated receptor-alpha (PPAR-α), reducing body weight and blood glucose [65]. Interestingly, they recently identified that bilirubin could bind directly to activate PPAR-α, which increased target genes to reduce adiposity [66,67].

Endothelial inflammation and dysfunction are key players in the initiation of atherosclerosis progression. The overexpression of endothelial HO-1 significantly attenuated the production of inflammatory mediators and improved the impaired vasodilatory responses of aortic segments treated with oxidized LDL [68]. Oxidized LDL increases the production of ROS, leading to NF-κB activation, which upregulates intercellular adhesion molecule (ICAM-1), vascular cell adhesion molecule (VCAM-1), and E-selectin expression in endothelial cells and increases the adhesion of monocytes [69]. The activation of Nrf2 was reported to suppress the endothelial cell activation by inactivating p38 MAPK activity, thereby suppressing VCAM-1 expression [70]. In vascular endothelium, atherosclerotic plaque is often observed in areas where disturbed blood flow is formed, whereas an atheroprotective region is found in areas where a steady laminar flow has developed. Kim et al. demonstrated that a laminar flow-induced activation of Nrf2 signaling pathway played a critical role in the anti-inflammatory and anti-apoptotic mechanisms in endothelial cells [71]. These observations thus suggest that the upregulation of HO-1 in vascular endothelial cells contributes significantly to the inhibition of atherosclerosis. Both cell-based and in vivo studies demonstrated that the induction of HO-1 protected the vessel walls from pathological remodeling and endothelial cell dysfunction [72,73]. In human endothelial cells, HO-1/CO also inhibited endoplasmic reticulum stress-induced apoptosis via p38 MAPK-dependent inhibition of the proapoptotic C/EBP homologous protein (CHOP) expression [74].

Atherosclerosis is regarded as a chronic inflammatory state in which macrophages play different and important roles. HO-1 in macrophages appears to be of critical importance for driving the resolution of inflammatory responses [75]. Orozco et al. reported that decreased HO-1 expression increased the expression of proinflammatory cytokines such as monocyte chemoattractant protein 1 (MCP-1) and interleukin 6 (IL-6) and the expression of scavenger receptor A (SR-A), and it also accelerated foam cell formation [76]. Ruotsalainen et al. [77] demonstrated that the peritoneal macrophages isolated from Nrf2-knockout (*Nfe2l2*^−/−^) mice showed increased expressions of MCP-1, IL-6, and tumor necrosis factor-alpha (TNF-α). With the stimulation of Nrf2^−/−^ peritoneal macrophages with oxidized LDL or lipopolysaccharide (LPS), the ROS production was increased with a concomitant induction of pro-inflammatory genes [78,79]. The anti-inflammatory effect of Nrf2 in macrophages is likely due to an improved antioxidant defense system. The cytoprotective action of bilirubin was reported to be partly related to its capacity to inhibit inducible NOS (iNOS), which leads to less production of the highly reactive and potent ONOO^−^ free radical [80]. Bilirubin inhibited iNOS expression and NO production in response to endotoxin in murine macrophages and in rats. CO also mediates part of the antioxidant and anti-inflammatory effects of HO-1. The increase of CO-exposure, whether produced endogenously from induction of HO-1 or delivered exogenously via a CO-releasing molecule, inhibited the LPS-derived upregulation of iNOS expression and NO overproduction in macrophages [81].

## 4. HO-1 Expression in Atherosclerotic Diseases States (Animal Studies)

### 4.1. Myocardial Infarction

HO-1 is suggested to be a meaningful player in the maintenance of cardiac homeostasis and the subsequent cardiac damage. Sharma et al. [82] demonstrated that ischemia/reperfusion substantially enhanced HO-1 expression in the porcine heart, suggesting a potential role of HO-1 in the defense against pathophysiological stress. In HO-1-deficient (*Hmox1*^−/−^) mice, hypoxia induced severe right ventricular dilatation and infarction [83]. The absence of *Hmox1* was reported to exacerbate ischemia/reperfusion-induced myocardial damage [84]. In contrast, a cardiac-specific overexpression of HO-1 reduced the myocardial infarct size and the inflammatory cell infiltration after ischemia/reperfusion [85]. The transfer of human HO-1 gene (*HMOX1*) before myocardial injury provided long-term myocardial protection from ischemia/reperfusion injury [86]. Tang et al. demonstrated that *HMOX1* gene transfer improved the contractile and diastolic performance after myocardial infarction in mice [87,88]. Issan et al. [89] reported that HO-1 induction by cobalt-protoporphyrin (CoPP) improved the cardiac function and decreased the infarct size in diabetic mice subjected to myocardial infarction. They also demonstrated that HO-1 induction increased the activity of the Akt prosurvival pathway in cardiomyocytes and decreased the plasma TNF-α level.

### 4.2. Heart Failure

In heart failure model mice produced by coronary ligation, myocyte-specific HO-1 overexpression improved the postinfarction survival and alleviated left ventricular remodeling; it also promoted neovascularization and ameliorated apoptosis [90]. Cardiac HO-1 overexpression could be either protective or detrimental in the heart depending on the type of stress context. Allwood et al. [91] demonstrated that cardiac-specific HO-1 overexpression significantly attenuated cardiac dysfunction, interstitial fibrosis, and hypertrophy induced by isoproterenol, whereas HO-1 had detrimental effects on the development of cardiomyopathic heart failure induced by pressure overload or aging.

## 5. HO-1 Expression in Patients with Atherosclerotic Diseases (Clinical Studies)

### 5.1. Carotid Atherosclerosis

**Figure 1:**
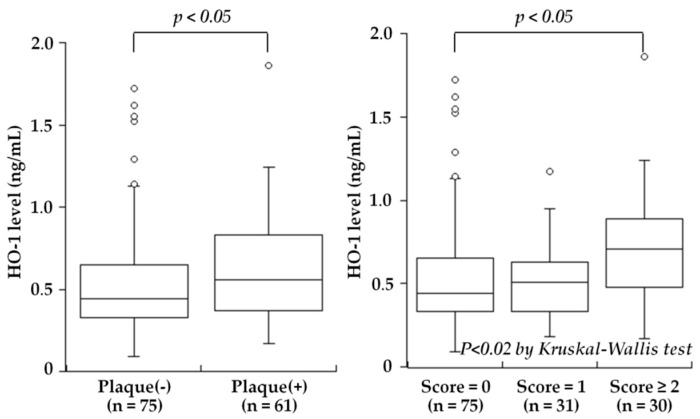
Plasma HO-1 levels and the presence of carotid plaque or the plaque score. Plasma HO-1 levels were significantly higher in subjects with carotid plaque than in those without plaque (*p* < 0.05) (**left**). A stepwise increase in HO-1 levels was found depending on the plaque score: 0.44 ng/mL in subjects with score = 0, 0.51 ng/mL in score = 1, and 0.70 ng/mL in score ≥ 2 (*p* < 0.02). HO-1 levels in score ≥ 2 were higher than those in score = 0 (*p* < 0.05) (**right**). The central line represents the median, and the box represents the 25th to 75th percentiles. The whiskers represent the lowest and highest value in the 25th percentile minus 1.5 IQR and 75th percentile plus 1.5 IQR, respectively. (Modified from Kishimoto et al. [25]).

One of the major problems in CAD is related to the significant length of time between the start of subclinical atherosclerosis and the manifestation of the disease, highlighting the importance of identifying biomarkers that can be used to predict CVD progression at as early a stage as possible. Elevated blood levels of HO-1 have been reported in chronic diseases such as diabetes mellitus [92], chronic silicosis [93], Parkinson’s disease [94], and hemophagocytic syndrome [95]. Although the precise mechanisms of secretion and the significance of the extracellular HO-1 remain to be determined, HO-1 is known to be released into the plasma by leukocytes, macrophages, smooth muscle cells, and endothelial cells that are activated or damaged by oxidative stress or inflammation [4]. Kishimoto et al. hypothesized that plasma HO-1 levels may be associated with the presence and severity of carotid atherosclerosis, and they measured the plasma HO-1 levels in 136 consecutive subjects (mean age 66 ± 9 years) who underwent carotid ultrasonography for a medical check-up to evaluate atherosclerosis [25]. The study was the first to reveal that the plasma HO-1 levels were significantly higher in the subjects with carotid plaque than in those without plaque (median 0.56 versus 0.44 ng/mL, *p* < 0.05), and the levels were stepwisely increased depending on the severity of plaque, defined as the plaque score (Figure 1). Moreover, the plasma HO-1 levels were significantly correlated with the plaque score (r = 0.23, *p* < 0.01 by Spearman’s rank correlation test). In a multivariate analysis, high HO-1 level (>0.50 ng/mL) was a significant factor associated with the presence of carotid plaque, independent of atherosclerotic risk factors (odds ratio: 2.33, 95% CI: 1.15–4.75, *p* < 0.025). Thus, the study reported plasma HO-1 levels to be high in subjects with carotid plaques and to be associated with the severity of carotid atherosclerosis. High plasma HO-1 levels may reflect an increased oxidative stress condition and may be aimed at protecting the body against the progression of atherosclerosis.

### 5.2. Coronary Artery Disease (CAD) and Peripheral Artery Disease (PAD)

CAD and PAD are chronic progressive atherosclerotic diseases leading to thrombosis and ischemia. CAD is the most common type of atherosclerotic diseases, followed by stroke and PAD [96]. PAD is a common circulatory problem in which narrowed arteries reduce the blood flow, mainly to the lower limbs. Many patients with PAD have mild or no symptoms, but some may have leg pain when walking. Patients with PAD often suffer from multiple arterial co-morbidities leading to high CVD-related mortality or a poor prognosis within a short time frame.

Cheng et al. [19] reported that the HO-1 expression in carotid endarterectomy samples was higher in unstable plaques than in stable plaques. They showed that the HO-1 level was positively correlated with features of vulnerable human atheromatous plaque, such as macrophage and lipid accumulation, and that the HO-1 level was inversely correlated with stable plaque features like the presence of intra-plaque smooth muscle cells and collagen. Yunoki et al. [22] also reported that the majority of HO-1-positive cells were macrophages, and the percentage of HO-1-positive areas was significantly higher in coronary atherectomy samples from patients with unstable angina pectoris (UAP) compared to those from patients with stable angina pectoris (SAP). Ijas et al. [17] reported that symptomatic plaques overexpressed HO-1 and CD163 which is involved in the degradation of hemoglobin and such expressions were correlated with traditional markers of unstable carotid disease, i.e., the degree of carotid stenosis and plaque ulcerations. In contrast, Ameriso et al. [16] investigated HO-1 expression in relation to *Helicobacter pylori* (*H. pylori*) infection and stated that HO-1 expression was more frequent in infected and asymptomatic carotid plaques. They suggested a potential role of *H. pylori* in oxidative stress-mediated injury and a subsequent defense reaction represented by HO-1 expression. In blood leukocytes, high HO-1 expression was observed by Chen et al. in patients with CAD, especially those with acute myocardial infarction (AMI) or UAP [15]. Chen et al. also showed that the mRNA expression of *HMOX1* in leukocytes was associated with the severity of CAD [97]. Brydun et al. [18] demonstrated that the capacity to upregulate *HMOX1* mRNA expression in leukocytes was inversely related to the degree of CAD. More recently, Fiorelli et al. [27] detected higher levels of HO-1 and Nrf2 in monocyte-derived macrophages (MDMs) of their CAD patients compared to those of healthy subjects. Of note, the patients with high levels of HO-1 more frequently displayed a thin cap fibroatheroma, a ruptured plaque, and the presence of thrombi.

**Figure 2:**
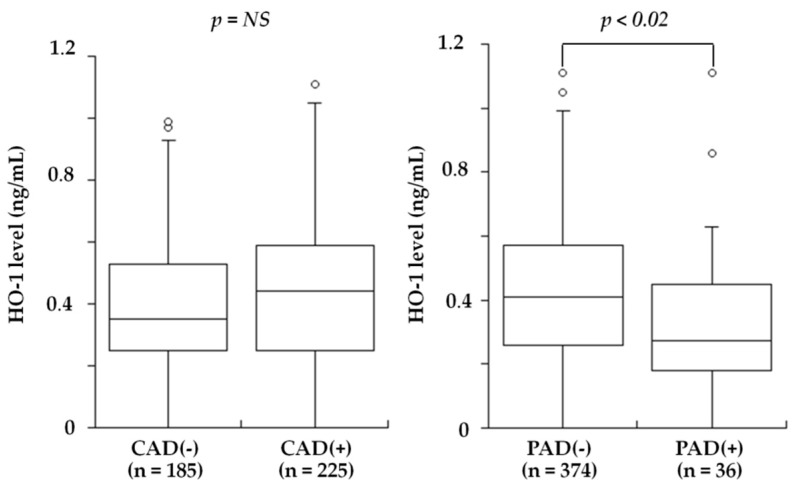
Plasma HO-1 levels and the presence of CAD or PAD. Plasma HO-1 levels tended to be higher in patients with CAD than in CAD(-) (median 0.44 versus 0.35 ng/mL), but this difference did not reach statistical significance (**left**). In contrast, HO-1 levels were significantly lower in patients with PAD than in PAD(-) (0.27 versus 0.41 ng/mL, *p* < 0.02) (**right**). The central line represents the median, and the box represents the 25th to 75th percentiles. The whiskers represent the lowest and highest value in the 25th percentile minus 1.5 IQR and 75th percentile plus 1.5 IQR, respectively. (Modified by Kishimoto et al. [26]).

**Figure 3:**
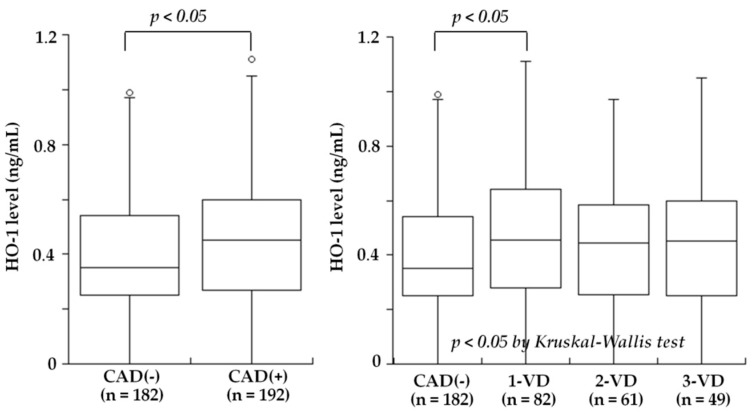
Plasma HO-1 levels and the presence of CAD or the number of stenotic coronary vessels among the 374 patients without PAD. After excluding the 36 patients with PAD, HO-1 levels were significantly higher in patients with CAD than in CAD(-) (median 0.45 versus 0.35 ng/mL, *p* < 0.05) (**left**). Furthermore, HO-1 levels in the 4 groups of CAD(-), 1-VD, 2-VD, and 3-VD were 0.35, 0.49, 0.44, and 0.44 ng/mL, respectively, and were highest in 1-VD (*p* < 0.05) (**right**). The central line represents the median, and the box represents the 25th to 75th percentiles. The whiskers represent the lowest and highest value in the 25th percentile minus 1.5 IQR and 75th percentile plus 1.5 IQR, respectively. (Modified by Kishimoto et al. [26]).

Several studies recently examined blood HO-1 levels in patients with atherosclerotic diseases. Idriss et al. [20] noted that the plasma HO-1 levels was raised in patients with stable CAD and increased further in those with acute coronary syndrome (ACS) compared to controls. Novo et al. [21] found that the serum HO-1 levels in patients with AMI were significantly higher compared to those of controls, and they revealed an inverse association with the severity of CAD. They also indicated that the HO-1 sequence was compatible with mechanisms of secretion and that therefore, its presence in the serum of patients might not necessarily be dependent on cell necrosis. Kishimoto et al. recently investigated the plasma HO-1 levels in 410 consecutive patients undergoing elective coronary angiography for suspected CAD who also had an ankle-brachial index (ABI) test to screen for PAD [26]. The plasma HO-1 levels did not differ between the patients with and without CAD (median 0.44 versus 0.35 ng/mL, *p* = NS). Notably, the HO-1 levels were significantly lower in the patients with PAD than in those without PAD (median 0.27 versus 0.41 ng/mL, *p* < 0.02) (Figure 2). However, the patients with PAD more often had CAD, especially three-vessel disease, compared to the patients without PAD (92% versus 51%, *p* < 0.01). After excluding the patients with PAD, the HO-1 levels were significantly higher in the patients with CAD than in those without CAD (0.45 versus 0.35 ng/mL, *p* < 0.05) and were highest in the patients with one-vessel disease among the four groups of CAD(-), one-vessel (1-VD), two-vessel (2-VD), and three-vessel disease (3-VD) (0.35, 0.49, 0.44, and 0.44 ng/mL, *p* < 0.05) (Figure 3). In a multivariate analysis, the odds ratios for CAD and PAD were 0.65 (95% CI: 0.42–0.99, *p* < 0.05) and 2.12 (95% CI: 1.03–4.37, *p* < 0.05) for low HO-1 level (<0.35 ng/mL), respectively. Therefore, plasma HO-1 levels were found to be low in patients with PAD, in contrast to high levels in patients with CAD. These results thus suggested that high plasma HO-1 levels in patients with CAD, especially one-vessel disease, may be aimed at protecting against the progression of CAD. In contrast, low plasma levels of HO-1 may be a marker reflecting the presence of PAD and may play a role in the development of PAD. This is in line with the results reported by Signorelli et al. [24], who noted that the serum HO-1 levels were lower in 27 patients with PAD compared to 27 controls. Although the mechanism of low plasma HO-1 levels in patients with PAD remains unclear, the HO-1 defensive response to oxidative stress was reported to be attenuated at advanced age [98] and at the late stage of diabetes mellitus [99]. A long duration of a severe stress condition may therefore cause some disruption of the HO-1 defense system. Gene and cell therapy with HO-1 were shown to be effective in animal models of limb ischemia [100,101]. Since patients with PAD have low HO-1 levels in blood, HO-1 inducers may be used to treat patients with PAD to inhibit the progression of PAD.

## 6. Conclusions

Taken together, the above studies’ results strongly support the notion that HO-1 plays a protective role against the progression of atherosclerotic diseases, such as CAD and PAD. In the pathogenesis of atherosclerosis, the ability of HO-1 to generate bilirubin, an anti-oxidant molecule and an agonist for PPAR-α, and CO, a vasodilator and an anti-inflammatory and antiapoptotic molecule, is thought to play important roles. Although the relevance of pharmacological or gene therapy with HO-1 to atherosclerotic disease in humans has yet to be established, the overall outcome of the preclinical studies carried out clearly points to HO-1 as a potential therapeutic target in atherosclerotic diseases. It is also of interest that the anti-atherogenic effects of statins (HMG-CoA reductase inhibitors) and fibrates (PPAR ligands) are partly mediated through HO-1 induction [102,103,104]. A number of natural antioxidant compounds contained in foods and plants, such as curcumin and caffeic acid phenethyl ester (polyphenols), and sulforaphane (isothiocyanates), have been demonstrated to be effective inducers of HO-1 and exert defensive actions against oxidative stress-related diseases. [105,106,107,108]. Importantly, further prospective studies are needed to determine the precise association between plasma HO-1 levels and the progression of carotid atherosclerosis as well as CAD and PAD.

## Figures and Tables

**Table 1:** Studies of the relationships between heme oxygenase (HO)-1 levels and atherosclerotic diseases.

Wang et al., 1998 [14]	Ascending and abdominal aortas	Patients undergoing surgery for CAD (*n* = 3) or abdominal aortic aneurysm (*n* = 5)	HO-1 was highly expressed in human atherosclerotic lesions
Chen et al., 2005 [15]	Blood leukocytes	Control (*n* = 30) SAP (*n* = 30) UAP (*n* = 40) AMI (*n* = 35)	HO-1 protein expression was higher in patients with CAD (AMI > UAP > SAP > Control)
Ameriso et al., 2005 [16]	Carotid endarterectomy specimens	Controls (*n* = 7) Patients with symptomatic plaques (*n* = 25) or asymptomatic plaques (*n* = 23)	HO-1 expression is highly prevalent in asymptomatic plaques
Ijas et al., 2007 [17]	Carotid plaques	(a) Patients with bilateral high-grade stenosis (one being symptomatic and the other asymptomatic) (*n* = 4) (b) Patients with ipsilateral stroke symptoms (*n* = 22) or without cerebrovascular symptoms (*n* = 18)	HO-1 and CD163 were overexpressed in symptomatic carotid plaques in both intra-individual and inter-individual comparison
Brydun et al., 2007 [18]	Blood mononuclear cells	110 patients undergoing coronary angiography	The capacity to upregulate *HMOX1* mRNA expression was inversely related to the degree of CAD
Cheng et al., 2009 [19]	Carotid endarterectomy specimens	112 CAD patients	HO-1 protein expression correlated with the vulnerability of atheromatous plaque
Idriss et al., 2010 [20]	Plasma	Healthy controls (*n* = 50) Stable CAD (*n* = 70) ACS (*n* = 24)	HO-1 levels were higher in stable CAD and ACS patients
Novo et al., 2011 [21]	Serum (or plasma)	Controls (*n* = 40) AMI (*n* = 40)	HO-1 levels in AMI patients were significantly higher than in controls, and showed an inverse association with the severity of CAD
Yunoki et al., 2013 [22]	Coronary atherectomy specimens	SAP (*n* = 33) UAP (*n* = 34)	HO-1-positive areas were significantly higher in UAP patients
Li et al., 2014 [23]	Serum	Stroke (*n* = 60) TIA (*n* = 50)	HO-1 levels were higher in patients with stroke than TIA
Signorelli et al., 2016 [24]	Serum	Controls (*n* = 27) PAD (*n* = 27)	HO-1 levels were lower in PAD patients
Kishimoto et al., 2018 [25]	Plasma	136 subjects undergoing carotid ultrasonography for medical check-up	HO-1 levels were high in subjects with carotid plaques
Kishimoto et al., 2018 [26]	Plasma	410 patients undergoing coronary angiography for suspected CAD	HO-1 levels were low in patients with PAD, in contrast to high levels in patients with CAD
Fiorelli et al., 2019 [27]	Monocyte-derived macrophages (MDMs)	Healthy controls (10) CAD patients undergoing coronary angiography (30)	HO-1 levels were higher in MDMs of CAD patients and were associated with rupture-prone coronary plaque

SAP, stable angina pectoris; UAP, unstable angina pectoris; AMI, acute myocardial infarction; CAD, coronary artery disease; ACS, acute coronary syndrome; PAD, peripheral artery disease; TIA, transient ischemic attack.

## Abbreviations

HO	Heme oxygenase
CO	Carbon monoxide
CAD	Coronary artery disease
PAD	Peripheral artery disease
LDL	Low-density lipoprotein
UGT1A1	UDP-glucuronosyltransferase 1A1
PPAR-α	Peroxisome proliferator-activated receptor-alpha
MCP-1	Monocyte chemoattractant protein-1
IL-6	Interleukin-6
SR-A	Scavenger receptor-A
AMI	Acute myocardial infarction
ABI	Ankle-brachial index
CI	Confidence interval
SAP	Stable angina pectoris
UAP	Unstable angina pectoris
ACS	Acute coronary syndrome
TIA	Transient ischemic attack

## References

[1] Libby P., Ridker P.M., Maseri A. (2002). Inflammation and atherosclerosis. Circulation.

[2] Immenschuh S., Vijayan V., Janciauskiene S., Gueler F. (2017). Heme as a target for therapeutic interventions. Front. Pharmacol..

[3] Ryter S.W., Alam J., Choi A.M. (2006). Heme oxygenase-1/carbon monoxide: From basic science to therapeutic applications. Physiol. Rev..

[4] Abraham N.G., Kappas A. (2008). Pharmacological and clinical aspects of heme oxygenase. Pharmacol. Rev..

[5] Fredenburgh L.E., Merz A.A., Cheng S. (2015). Haeme oxygenase signalling pathway: Implications for cardiovascular disease. Eur. Heart J..

[6] Maines M.D. (1997). The heme oxygenase system: A regulator of second messenger gases. Ann. Rev. Pharmacol. Toxicol..

[7] Wu M.L., Ho Y.C., Lin C.Y., Yet S.F. (2011). Heme oxygenase-1 in inflammation and cardiovascular disease. Am. J. Cardiovasc. Dis..

[8] Yoshida T., Biro P., Cohen T., Muller R.M., Shibahara S. (1988). Human heme oxygenase cdna and induction of its mrna by hemin. Eur. J. Biochem..

[9] Alam J., Shibahara S., Smith A. (1989). Transcriptional activation of the heme oxygenase gene by heme and cadmium in mouse hepatoma cells. J. Biol. Chem..

[10] Keyse S.M., Tyrrell R.M. (1990). Induction of the heme oxygenase gene in human skin fibroblasts by hydrogen peroxide and uva (365 nm) radiation: Evidence for the involvement of the hydroxyl radical. Carcinogenesis.

[11] Cantoni L., Rossi C., Rizzardini M., Gadina M., Ghezzi P. (1991). Interleukin-1 and tumour necrosis factor induce hepatic haem oxygenase. Feedback regulation by glucocorticoids. Biochem. J..

[12] Rizzardini M., Carelli M., Cabello Porras M.R., Cantoni L. (1994). Mechanisms of endotoxin-induced haem oxygenase mrna accumulation in mouse liver: Synergism by glutathione depletion and protection by n-acetylcysteine. Biochem. J..

[13] Hayashi S., Omata Y., Sakamoto H., Higashimoto Y., Hara T., Sagara Y., Noguchi M. (2004). Characterization of rat heme oxygenase-3 gene. Implication of processed pseudogenes derived from heme oxygenase-2 gene. Gene.

[14] Wang L.J., Lee T.S., Lee F.Y., Pai R.C., Chau L.Y. (1998). Expression of heme oxygenase-1 in atherosclerotic lesions. Am. J. Pathol..

[15] Chen S.M., Li Y.G., Wang D.M. (2005). Study on changes of heme oxygenase-1 expression in patients with coronary heart disease. Clin. Cardiol..

[16] Ameriso S.F., Villamil A.R., Zedda C., Parodi J.C., Garrido S., Sarchi M.I., Schultz M., Boczkowski J., Sevlever G.E. (2005). Heme oxygenase-1 is expressed in carotid atherosclerotic plaques infected by helicobacter pylori and is more prevalent in asymptomatic subjects. Stroke.

[17] Ijas P., Nuotio K., Saksi J., Soinne L., Saimanen E., Karjalainen-Lindsberg M.L., Salonen O., Sarna S., Tuimala J., Kovanen P.T. (2007). Microarray analysis reveals overexpression of cd163 and ho-1 in symptomatic carotid plaques. Arterioscler. Thromb Vasc. Biol.

[18] Brydun A., Watari Y., Yamamoto Y., Okuhara K., Teragawa H., Kono F., Chayama K., Oshima T., Ozono R. (2007). Reduced expression of heme oxygenase-1 in patients with coronary atherosclerosis. Hypertens. Res..

[19] Cheng C., Noordeloos A.M., Jeney V., Soares M.P., Moll F., Pasterkamp G., Serruys P.W., Duckers H.J. (2009). Heme oxygenase 1 determines atherosclerotic lesion progression into a vulnerable plaque. Circulation.

[20] Idriss N.K., Lip G.Y., Balakrishnan B., Jaumdally R., Boos C.J., Blann A.D. (2010). Plasma haemoxygenase-1 in coronary artery disease. A comparison with angiogenin, matrix metalloproteinase-9, tissue inhibitor of metalloproteinase-1 and vascular endothelial growth factor. Thromb. Haemost..

[21] Novo G., Cappello F., Rizzo M., Fazio G., Zambuto S., Tortorici E., Marino Gammazza A., Corrao S., Zummo G., De Macario E.C. (2011). Hsp60 and heme oxygenase-1 (hsp32) in acute myocardial infarction. Transl. Res..

[22] Yunoki K., Inoue T., Sugioka K., Nakagawa M., Inaba M., Wada S., Ohsawa M., Komatsu R., Itoh A., Haze K. (2013). Association between hemoglobin scavenger receptor and heme oxygenase-1-related anti-inflammatory mediators in human coronary stable and unstable plaques. Hum. Pathol..

[23] Li X., Song G., Jin Y., Liu H., Li C., Han C., Ren S. (2014). Higher level of heme oxygenase-1 in patients with stroke than tia. J. Thorac. Dis..

[24] Signorelli S.S., Li Volsi G., Fiore V., Mangiafico M., Barbagallo I., Parenti R., Rizzo M., Li Volti G. (2016). Plasma heme oxygenase-1 is decreased in peripheral artery disease patients. Mol. Med. Rep..

[25] Kishimoto Y., Sasaki K., Saita E., Niki H., Ohmori R., Kondo K., Momiyama Y. (2018). Plasma heme oxygenase-1 levels and carotid atherosclerosis. Stroke.

[26] Kishimoto Y., Ibe S., Saita E., Sasaki K., Niki H., Miura K., Ikegami Y., Ohmori R., Kondo K., Momiyama Y. (2018). Plasma heme oxygenase-1 levels in patients with coronary and peripheral artery diseases. Dis. Markers.

[27] Fiorelli S., Porro B., Cosentino N., Di Minno A., Manega C.M., Fabbiocchi F., Niccoli G., Fracassi F., Barbieri S., Marenzi G. (2019). Activation of nrf2/ho-1 pathway and human atherosclerotic plaque vulnerability:An in vitro and in vivo study. Cells.

[28] Yachie A., Niida Y., Wada T., Igarashi N., Kaneda H., Toma T., Ohta K., Kasahara Y., Koizumi S. (1999). Oxidative stress causes enhanced endothelial cell injury in human heme oxygenase-1 deficiency. J. Clin. Investig..

[29] Radhakrishnan N., Yadav S.P., Sachdeva A., Pruthi P.K., Sawhney S., Piplani T., Wada T., Yachie A. (2011). Human heme oxygenase-1 deficiency presenting with hemolysis, nephritis, and asplenia. J. Pediatr. Hematol. Oncol..

[30] Poss K.D., Tonegawa S. (1997). Reduced stress defense in heme oxygenase 1-deficient cells. Proc. Natl. Acad. Sci. USA.

[31] Poss K.D., Tonegawa S. (1997). Heme oxygenase 1 is required for mammalian iron reutilization. Proc. Natl. Acad. Sci. USA.

[32] Ishikawa K., Navab M., Lusis A.J. (2012). Vasculitis, atherosclerosis, and altered hdl composition in heme-oxygenase-1-knockout mice. Int. J. Hypertens..

[33] Agarwal A., Balla J., Balla G., Croatt A.J., Vercellotti G.M., Nath K.A. (1996). Renal tubular epithelial cells mimic endothelial cells upon exposure to oxidized ldl. Am. J. Physiol..

[34] Yamaguchi M., Sato H., Bannai S. (1993). Induction of stress proteins in mouse peritoneal macrophages by oxidized low-density lipoprotein. Biochem. Biophys. Res. Commun..

[35] Ishikawa K., Navab M., Leitinger N., Fogelman A.M., Lusis A.J. (1997). Induction of heme oxygenase-1 inhibits the monocyte transmigration induced by mildly oxidized ldl. J. Clin. Investig..

[36] Ishikawa K., Sugawara D., Wang X., Suzuki K., Itabe H., Maruyama Y., Lusis A.J. (2001). Heme oxygenase-1 inhibits atherosclerotic lesion formation in ldl-receptor knockout mice. Circ. Res..

[37] Ishikawa K., Sugawara D., Goto J., Watanabe Y., Kawamura K., Shiomi M., Itabe H., Maruyama Y. (2001). Heme oxygenase-1 inhibits atherogenesis in watanabe heritable hyperlipidemic rabbits. Circulation.

[38] Juan S.H., Lee T.S., Tseng K.W., Liou J.Y., Shyue S.K., Wu K.K., Chau L.Y. (2001). Adenovirus-mediated heme oxygenase-1 gene transfer inhibits the development of atherosclerosis in apolipoprotein e-deficient mice. Circulation.

[39] Yet S.F., Layne M.D., Liu X., Chen Y.H., Ith B., Sibinga N.E., Perrella M.A. (2003). Absence of heme oxygenase-1 exacerbates atherosclerotic lesion formation and vascular remodeling. FASEB J..

[40] Qiao H., Sai X., Gai L., Huang G., Chen X., Tu X., Ding Z. (2014). Association between heme oxygenase 1 gene promoter polymorphisms and susceptibility to coronary artery disease: A huge review and meta-analysis. Am. J. Epidemiol..

[41] Yamada N., Yamaya M., Okinaga S., Nakayama K., Sekizawa K., Shibahara S., Sasaki H. (2000). Microsatellite polymorphism in the heme oxygenase-1 gene promoter is associated with susceptibility to emphysema. Am. J. Hum. Genet..

[42] Ono K., Goto Y., Takagi S., Baba S., Tago N., Nonogi H., Iwai N. (2004). A promoter variant of the heme oxygenase-1 gene may reduce the incidence of ischemic heart disease in japanese. Atherosclerosis.

[43] Schillinger M., Exner M., Mlekusch W., Domanovits H., Huber K., Mannhalter C., Wagner O., Minar E. (2002). Heme oxygenase-1 gene promoter polymorphism is associated with abdominal aortic aneurysm. Thromb. Res..

[44] Kaneda H., Ohno M., Taguchi J., Togo M., Hashimoto H., Ogasawara K., Aizawa T., Ishizaka N., Nagai R. (2002). Heme oxygenase-1 gene promoter polymorphism is associated with coronary artery disease in japanese patients with coronary risk factors. Arterioscler. Thromb. Vasc. Biol..

[45] Funk M., Endler G., Schillinger M., Mustafa S., Hsieh K., Exner M., Lalouschek W., Mannhalter C., Wagner O. (2004). The effect of a promoter polymorphism in the heme oxygenase-1 gene on the risk of ischaemic cerebrovascular events: The influence of other vascular risk factors. Thromb. Res..

[46] Chen Y.H., Chau L.Y., Chen J.W., Lin S.J. (2008). Serum bilirubin and ferritin levels link heme oxygenase-1 gene promoter polymorphism and susceptibility to coronary artery disease in diabetic patients. Diabetes Care.

[47] Chen M., Zhou L., Ding H., Huang S., He M., Zhang X., Cheng L., Wang D., Hu F.B., Wu T. (2012). Short (gt) ( n ) repeats in heme oxygenase-1 gene promoter are associated with lower risk of coronary heart disease in subjects with high levels of oxidative stress. Cell Stress Chaperones.

[48] Itoh K., Chiba T., Takahashi S., Ishii T., Igarashi K., Katoh Y., Oyake T., Hayashi N., Satoh K., Hatayama I. (1997). An nrf2/small maf heterodimer mediates the induction of phase ii detoxifying enzyme genes through antioxidant response elements. Biochem. Biophys. Res. Commun..

[49] Ooi B.K., Goh B.H., Yap W.H. (2017). Oxidative stress in cardiovascular diseases: Involvement of nrf2 antioxidant redox signaling in macrophage foam cells formation. Int. J. Mol. Sci..

[50] Sivandzade F., Prasad S., Bhalerao A., Cucullo L. (2019). Nrf2 and nf-b interplay in cerebrovascular and neurodegenerative disorders: Molecular mechanisms and possible therapeutic approaches. Redox Biol..

[51] Stocker R., Yamamoto Y., McDonagh A.F., Glazer A.N., Ames B.N. (1987). Bilirubin is an antioxidant of possible physiological importance. Science.

[52] Mayer M. (2000). Association of serum bilirubin concentration with risk of coronary artery disease. Clin. Chem..

[53] Boon A.C., Hawkins C.L., Bisht K., Coombes J.S., Bakrania B., Wagner K.H., Bulmer A.C. (2012). Reduced circulating oxidized ldl is associated with hypocholesterolemia and enhanced thiol status in gilbert syndrome. Free Radic. Biol. Med..

[54] Stojanov M., Stefanovic A., Dzingalasevic G., Ivanisevic J., Miljkovic M., Mandic-Radic S., Prostran M. (2013). Total bilirubin in young men and women: Association with risk markers for cardiovascular diseases. Clin. Biochem..

[55] Nascimento H., Alves A.I., Coimbra S., Catarino C., Gomes D., Bronze-da-Rocha E., Costa E., Rocha-Pereira P., Aires L., Mota J. (2015). Bilirubin is independently associated with oxidized ldl levels in young obese patients. Diabetol. Metab. Syndr..

[56] Schwertner H.A., Jackson W.G., Tolan G. (1994). Association of low serum concentration of bilirubin with increased risk of coronary artery disease. Clin. Chem..

[57] Turfan M., Duran M., Poyraz F., Yayla C., Akboga M.K., Sahinarslan A., Tavil Y., Pasaoglu H., Boyaci B. (2013). Inverse relationship between serum total bilirubin levels and severity of disease in patients with stable coronary artery disease. Coron. Artery Dis..

[58] Kang S.J., Kim D., Park H.E., Chung G.E., Choi S.H., Choi S.Y., Lee W., Kim J.S., Cho S.H. (2013). Elevated serum bilirubin levels are inversely associated with coronary artery atherosclerosis. Atherosclerosis.

[59] Akboga M.K., Canpolat U., Sahinarslan A., Alsancak Y., Nurkoc S., Aras D., Aydogdu S., Abaci A. (2015). Association of serum total bilirubin level with severity of coronary atherosclerosis is linked to systemic inflammation. Atherosclerosis.

[60] Lin J.P., Vitek L., Schwertner H.A. (2010). Serum bilirubin and genes controlling bilirubin concentrations as biomarkers for cardiovascular disease. Clin. Chem..

[61] Stender S., Frikke-Schmidt R., Nordestgaard B.G., Grande P., Tybjaerg-Hansen A. (2013). Genetically elevated bilirubin and risk of ischaemic heart disease: Three mendelian randomization studies and a meta-analysis. J. Intern. Med..

[62] Kobayashi A., Ishikawa K., Matsumoto H., Kimura S., Kamiyama Y., Maruyama Y. (2007). Synergetic antioxidant and vasodilatory action of carbon monoxide in angiotensin ii - induced cardiac hypertrophy. Hypertension.

[63] Taille C., El-Benna J., Lanone S., Dang M.C., Ogier-Denis E., Aubier M., Boczkowski J. (2004). Induction of heme oxygenase-1 inhibits nad(p)h oxidase activity by down-regulating cytochrome b558 expression via the reduction of heme availability. J. Biol. Chem..

[64] Abraham N.G., Junge J.M., Drummond G.S. (2016). Translational significance of heme oxygenase in obesity and metabolic syndrome. Trends Pharmacol. Sci..

[65] Hinds T.D., Sodhi K., Meadows C., Fedorova L., Puri N., Kim D.H., Peterson S.J., Shapiro J., Abraham N.G., Kappas A. (2014). Increased ho-1 levels ameliorate fatty liver development through a reduction of heme and recruitment of fgf21. Obesity (Silver Spring).

[66] Stec D.E., John K., Trabbic C.J., Luniwal A., Hankins M.W., Baum J., Hinds T.D. (2016). Bilirubin binding to pparalpha inhibits lipid accumulation. PLoS ONE.

[67] Gordon D.M., Blomquist T.M., Miruzzi S.A., McCullumsmith R., Stec D.E., Hinds T.D. (2019). Rna sequencing in human hepg2 hepatocytes reveals ppar-alpha mediates transcriptome responsiveness of bilirubin. Physiol. Genom..

[68] Kawamura K., Ishikawa K., Wada Y., Kimura S., Matsumoto H., Kohro T., Itabe H., Kodama T., Maruyama Y. (2005). Bilirubin from heme oxygenase-1 attenuates vascular endothelial activation and dysfunction. Arterioscler. Thromb. Vasc. Biol..

[69] Kim J.A., Territo M.C., Wayner E., Carlos T.M., Parhami F., Smith C.W., Haberland M.E., Fogelman A.M., Berliner J.A. (1994). Partial characterization of leukocyte binding molecules on endothelial cells induced by minimally oxidized ldl. Arterioscler. Thromb..

[70] Zakkar M., Van der Heiden K., Luong le A., Chaudhury H., Cuhlmann S., Hamdulay S.S., Krams R., Edirisinghe I., Rahman I., Carlsen H. (2009). Activation of nrf2 in endothelial cells protects arteries from exhibiting a proinflammatory state. Arterioscler. Thromb. Vasc. Biol..

[71] Kim M., Kim S., Lim J.H., Lee C., Choi H.C., Woo C.H. (2012). Laminar flow activation of erk5 protein in vascular endothelium leads to atheroprotective effect via nf-e2-related factor 2 (nrf2) activation. J. Biol. Chem..

[72] Duckers H.J., Boehm M., True A.L., Yet S.F., San H., Park J.L., Clinton Webb R., Lee M.E., Nabel G.J., Nabel E.G. (2001). Heme oxygenase-1 protects against vascular constriction and proliferation. Nat. Med..

[73] Li T., Tian H., Zhao Y., An F., Zhang L., Zhang J., Peng J., Zhang Y., Guo Y. (2011). Heme oxygenase-1 inhibits progression and destabilization of vulnerable plaques in a rabbit model of atherosclerosis. Eur. J. Pharmacol..

[74] Kim K.M., Pae H.O., Zheng M., Park R., Kim Y.M., Chung H.T. (2007). Carbon monoxide induces heme oxygenase-1 via activation of protein kinase r-like endoplasmic reticulum kinase and inhibits endothelial cell apoptosis triggered by endoplasmic reticulum stress. Circ. Res..

[75] Vijayan V., Wagener F., Immenschuh S. (2018). The macrophage heme-heme oxygenase-1 system and its role in inflammation. Biochem. Pharmacol..

[76] Orozco L.D., Kapturczak M.H., Barajas B., Wang X., Weinstein M.M., Wong J., Deshane J., Bolisetty S., Shaposhnik Z., Shih D.M. (2007). Heme oxygenase-1 expression in macrophages plays a beneficial role in atherosclerosis. Circ. Res..

[77] Ruotsalainen A.K., Inkala M., Partanen M.E., Lappalainen J.P., Kansanen E., Makinen P.I., Heinonen S.E., Laitinen H.M., Heikkila J., Vatanen T. (2013). The absence of macrophage nrf2 promotes early atherogenesis. Cardiovasc. Res..

[78] Barajas B., Che N., Yin F., Rowshanrad A., Orozco L.D., Gong K.W., Wang X., Castellani L.W., Reue K., Lusis A.J. (2011). Nf-e2-related factor 2 promotes atherosclerosis by effects on plasma lipoproteins and cholesterol transport that overshadow antioxidant protection. Arterioscler. Thromb. Vasc. Biol..

[79] Thimmulappa R.K., Scollick C., Traore K., Yates M., Trush M.A., Liby K.T., Sporn M.B., Yamamoto M., Kensler T.W., Biswal S. (2006). Nrf2-dependent protection from lps induced inflammatory response and mortality by cddo-imidazolide. Biochem. Biophys. Res. Commun..

[80] Wang W.W., Smith D.L., Zucker S.D. (2004). Bilirubin inhibits inos expression and no production in response to endotoxin in rats. Hepatology.

[81] Srisook K., Han S.S., Choi H.S., Li M.H., Ueda H., Kim C., Cha Y.N. (2006). Co from enhanced ho activity or from corm-2 inhibits both o2- and no production and downregulates ho-1 expression in lps-stimulated macrophages. Biochem. Pharmacol..

[82] Sharma H.S., Maulik N., Gho B.C., Das D.K., Verdouw P.D. (1996). Coordinated expression of heme oxygenase-1 and ubiquitin in the porcine heart subjected to ischemia and reperfusion. Mol. Cell Biochem..

[83] Yet S.F., Perrella M.A., Layne M.D., Hsieh C.M., Maemura K., Kobzik L., Wiesel P., Christou H., Kourembanas S., Lee M.E. (1999). Hypoxia induces severe right ventricular dilatation and infarction in heme oxygenase-1 null mice. J. Clin. Investig..

[84] Liu X., Wei J., Peng D.H., Layne M.D., Yet S.F. (2005). Absence of heme oxygenase-1 exacerbates myocardial ischemia/reperfusion injury in diabetic mice. Diabetes.

[85] Yet S.F., Tian R., Layne M.D., Wang Z.Y., Maemura K., Solovyeva M., Ith B., Melo L.G., Zhang L., Ingwall J.S. (2001). Cardiac-specific expression of heme oxygenase-1 protects against ischemia and reperfusion injury in transgenic mice. Circ. Res..

[86] Melo L.G., Agrawal R., Zhang L., Rezvani M., Mangi A.A., Ehsan A., Griese D.P., Dell’Acqua G., Mann M.J., Oyama J. (2002). Gene therapy strategy for long-term myocardial protection using adeno-associated virus-mediated delivery of heme oxygenase gene. Circulation.

[87] Tang Y.L., Tang Y., Zhang Y.C., Qian K., Shen L., Phillips M.I. (2004). Protection from ischemic heart injury by a vigilant heme oxygenase-1 plasmid system. Hypertension.

[88] Tang Y.L., Qian K., Zhang Y.C., Shen L., Phillips M.I. (2005). A vigilant, hypoxia-regulated heme oxygenase-1 gene vector in the heart limits cardiac injury after ischemia-reperfusion in vivo. J. Cardiovasc. Pharmacol. Ther..

[89] Issan Y., Kornowski R., Aravot D., Shainberg A., Laniado-Schwartzman M., Sodhi K., Abraham N.G., Hochhauser E. (2014). Heme oxygenase-1 induction improves cardiac function following myocardial ischemia by reducing oxidative stress. PLoS ONE.

[90] Wang G., Hamid T., Keith R.J., Zhou G., Partridge C.R., Xiang X., Kingery J.R., Lewis R.K., Li Q., Rokosh D.G. (2010). Cardioprotective and antiapoptotic effects of heme oxygenase-1 in the failing heart. Circulation.

[91] Allwood M.A., Kinobe R.T., Ballantyne L., Romanova N., Melo L.G., Ward C.A., Brunt K.R., Simpson J.A. (2014). Heme oxygenase-1 overexpression exacerbates heart failure with aging and pressure overload but is protective against isoproterenol-induced cardiomyopathy in mice. Cardiovasc. Pathol..

[92] Bao W., Song F., Li X., Rong S., Yang W., Zhang M., Yao P., Hao L., Yang N., Hu F.B. (2010). Plasma heme oxygenase-1 concentration is elevated in individuals with type 2 diabetes mellitus. PLoS ONE.

[93] Sato T., Takeno M., Honma K., Yamauchi H., Saito Y., Sasaki T., Morikubo H., Nagashima Y., Takagi S., Yamanaka K. (2006). Heme oxygenase-1, a potential biomarker of chronic silicosis, attenuates silica-induced lung injury. Am. J. Respir. Crit. Care Med..

[94] Mateo I., Infante J., Sanchez-Juan P., Garcia-Gorostiaga I., Rodriguez-Rodriguez E., Vazquez-Higuera J.L., Berciano J., Combarros O. (2010). Serum heme oxygenase-1 levels are increased in parkinson’s disease but not in alzheimer’s disease. Acta Neurol. Scand..

[95] Miyazaki T., Kirino Y., Takeno M., Hama M., Ushihama A., Watanabe R., Takase K., Tachibana T., Matsumoto K., Tanaka M. (2010). Serum ho-1 is useful to make differential diagnosis of secondary hemophagocytic syndrome from other similar hematological conditions. Int. J. Hematol..

[96] Fowkes F.G., Rudan D., Rudan I., Aboyans V., Denenberg J.O., McDermott M.M., Norman P.E., Sampson U.K., Williams L.J., Mensah G.A. (2013). Comparison of global estimates of prevalence and risk factors for peripheral artery disease in 2000 and 2010: A systematic review and analysis. Lancet.

[97] Chen S.M., Li Y.G., Wang D.M., Zhang G.H., Tan C.J. (2009). Expression of heme oxygenase-1, hypoxia inducible factor-1alpha, and ubiquitin in peripheral inflammatory cells from patients with coronary heart disease. Clin. Chem. Lab. Med..

[98] Secher N., Ostergaard L., Tonnesen E., Hansen F.B., Granfeldt A. (2018). Impact of age on cardiovascular function, inflammation, and oxidative stress in experimental asphyxial cardiac arrest. Acta Anaesthesiol. Scand..

[99] Song F., Qi X., Chen W., Jia W., Yao P., Nussler A.K., Sun X., Liu L. (2007). Effect of momordica grosvenori on oxidative stress pathways in renal mitochondria of normal and alloxan-induced diabetic mice. Involvement of heme oxygenase-1. Eur. J. Nutr..

[100] Suzuki M., Iso-o N., Takeshita S., Tsukamoto K., Mori I., Sato T., Ohno M., Nagai R., Ishizaka N. (2003). Facilitated angiogenesis induced by heme oxygenase-1 gene transfer in a rat model of hindlimb ischemia. Biochem. Biophys. Res. Commun..

[101] Grochot-Przeczek A., Kotlinowski J., Kozakowska M., Starowicz K., Jagodzinska J., Stachurska A., Volger O.L., Bukowska-Strakova K., Florczyk U., Tertil M. (2014). Heme oxygenase-1 is required for angiogenic function of bone marrow-derived progenitor cells: Role in therapeutic revascularization. Antioxid. Redox Signal..

[102] Lee T.S., Chang C.C., Zhu Y., Shyy J.Y. (2004). Simvastatin induces heme oxygenase-1: A novel mechanism of vessel protection. Circulation.

[103] Heeba G., Moselhy M.E., Hassan M., Khalifa M., Gryglewski R., Malinski T. (2009). Anti-atherogenic effect of statins: Role of nitric oxide, peroxynitrite and haem oxygenase-1. Br. J. Pharmacol..

[104] Wang Y., Yu M., Ma Y., Wang R., Liu W., Xia W., Guan A., Xing C., Lu F., Ji X. (2017). Fenofibrate increases heme oxygenase 1 expression and astrocyte proliferation while limits neuronal injury during intracerebral hemorrhage. Curr. Neurovasc. Res..

[105] Li Volti G., Sacerdoti D., Di Giacomo C., Barcellona M.L., Scacco A., Murabito P., Biondi A., Basile F., Gazzolo D., Abella R. (2008). Natural heme oxygenase-1 inducers in hepatobiliary function. World J. Gastroenterol..

[106] Pittala V., Vanella L., Salerno L., Romeo G., Marrazzo A., Di Giacomo C., Sorrenti V. (2018). Effects of polyphenolic derivatives on heme oxygenase-system in metabolic dysfunctions. Curr. Med. Chem..

[107] Pittala V., Vanella L., Salerno L., Di Giacomo C., Acquaviva R., Raffaele M., Romeo G., Modica M.N., Prezzavento O., Sorrenti V. (2017). Novel caffeic acid phenethyl ester (cape) analogues as inducers of heme oxygenase-1. Curr. Pharm Des..

[108] Pittala V., Salerno L., Romeo G., Acquaviva R., Di Giacomo C., Sorrenti V. (2018). Therapeutic potential of caffeic acid phenethyl ester (cape) in diabetes. Curr. Med. Chem..

